# The role of the microbiota-gut-brain axis in schizophrenia: an immunological perspective

**DOI:** 10.3389/fimmu.2025.1711756

**Published:** 2025-11-19

**Authors:** Bo-Wei Su, Yao Li, Le-Ying Yang, Hai-Xia Yang, Wen-Hao Wang, Hui-Wen Ren, Ya-Nan Bao, Jia-Yi Lao, Zhi-Lin Luan

**Affiliations:** 1 Advanced Institute for Medical Sciences, Dalian Medical University, Dalian, China; 2 Department of Neuroregulation Center, Southwest Rehabilitation Hospital, Chengdu, China; 3 School of Education, University of New South Wales, Sydney, NSW, Australia; 4 Dalian Key Laboratory for Nuclear Receptors in Major Metabolic Diseases, Dalian, China

**Keywords:** schizophrenia, microbiota-gut-brain axis, gut microbiota metabolites, immune dysregulation, neuroinflammation, kynurenine pathway, complement system, toll-like receptors

## Abstract

Schizophrenia (SZ) is a severe neuropsychiatric disorder arising from complex interactions between genetic susceptibility and environmental factors. There is growing evidence that immune dysregulation and neuroinflammation are central to its pathogenesis, with the microbiota-gut-brain (MGB) axis playing a critical role. This review synthesizes clinical and preclinical findings to elucidate the relationship between gut microbiota dysbiosis and aberrant inflammatory signaling in the periphery and central nervous system in schizophrenia. We detail how alterations in gut microbiota metabolites, following dysbiosis disrupt blood-brain barrier (BBB) integrity and exacerbate neuroinflammation, ultimately leading to the neuropathology of SZ. The review further explores how gut dysbiosis activates innate immune pathways, including the complement system (e.g., C4) and Toll-like receptors (e.g., TLR4), and examines the bidirectional relationship between cytokine imbalances and gut microbiota. A key focus is placed on the dysregulation of the kynurenine pathway of tryptophan metabolism, which mechanistically links immune activation to neurotransmitter imbalances. Collectively, these findings demonstrate that gut microbiota dysbiosis contributes to the pathophysiology of schizophrenia through multifaceted immune-neuro-endocrine pathways, highlighting the MGB axis as a promising target for novel therapeutic strategies.

## 1 Introduction

Schizophrenia (SZ) is a chronic brain disorder characterized by genetic heterogeneity and neuropathological alterations, with high mortality rates (1). It impairs higher-order brain functions, leading to multifaceted disabilities and incoordination of mental activities. Its clinical symptoms primarily include positive symptoms, negative symptoms, and cognitive deficits (2, 3). Evidence supports the interplay between genetic and environmental factors plays a crucial role in the pathogenesis of schizophrenia (4–6).

**Figure 1:**
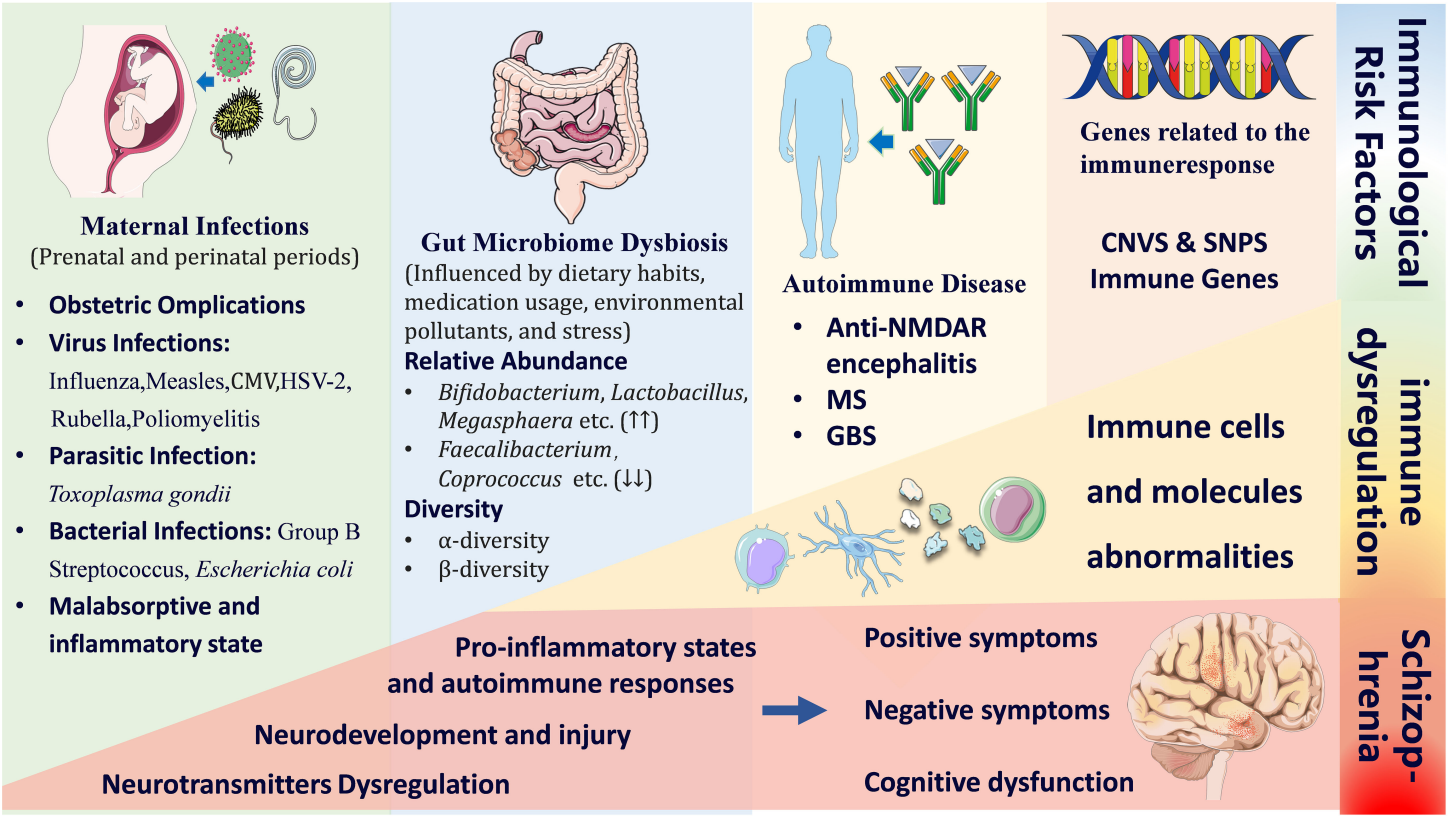
Immune-related risk factors that may induce schizophrenia and pathophysiological consequences. The figure provides an overview of the process by which immune-related risk factors disrupt the balance of the immune system, ultimately contributing to the development of SZ. The risk factors associated with immune system imbalance include intrinsic factors (such as autoimmune diseases) and extrinsic environmental factors (such as maternal infections, environmental exposures during early life, childhood, and the first episode of psychosis, as well as gut microbiota dysbiosis). Immune system imbalance is primarily reflected in abnormalities of immune cells, immune organs, and immune-related molecules. The pathological mechanisms by which immune system imbalance may lead to SZ include neurotransmitter dysregulation (abnormalities in dopamine/glutamate/GABA/5-HT), neurodevelopmental impairments (such as abnormal synaptic pruning and brain structural changes), inflammatory responses, autoimmune reactions, and related clinical symptoms (positive symptoms, negative symptoms, and cognitive deficits). CMV, Cytomegalovirus; HSV-2, Herpes Simplex Virus Type 2; MS, Multiple Sclerosis; GBS, Guillain-Barré Syndrome; CNVs, Copy Number Variations; SNPs, Single Nucleotide Polymorphism.

Current evidence from genetics, molecular neuropathology, and clinical studies underscores the importance of immune factors in both the pathogenesis and treatment of schizophrenia (7–9). Notably, genetic data from multiple large-scale patient cohorts and genome-wide association studies have shown that single nucleotide polymorphisms located within the major immune-related region on chromosome 6 are associated with schizophrenia (10–12). This finding provides strong genetic support for the immune hypothesis of schizophrenia. Furthermore, immune dysfunction may indirectly increase the risk of developing schizophrenia (see **Figure 1**). This dysfunction can be triggered by intrinsic host factors, such as autoimmune diseases (13–15), as well as extrinsic environmental factors, such as maternal infection and environmental exposures to infections during early life, childhood, and around the first episode of psychosis (16, 17).

Among these risk factors, infectious pathogens play a significant role as environmental risk factors in SZ. Clinical evidence (see **Table 1**) and meta-analyses (30) show a significant association between schizophrenia and a history of infection with various pathogens (for example, *Chlamydophila psittaci*, *Chlamydia pneumoniae*, Human Herpesvirus 2, Borna disease virus, and Human Endogenous Retrovirus W). Exposure to these infectious pathogens and inflammatory stimuli can profoundly affect the brain and behavior (31). A prominent example is *Toxoplasma gondii* infection, which, in animal models, alters behavior and increases the release of neurotransmitters such as dopamine, and the amount of dopamine release is correlated with the number of infected cells (32). In humans, it is associated with symptoms similar to those observed in schizophrenia (33). Notably, such infections can induce a complex neuroimmune response involving cytokine production by microglia, astrocytes, and neurons (34). Furthermore, they can dysregulate key immune components; for example, CD8^+^T cells, which are crucial for sustaining lasting immunity, have been found to be downregulated in schizophrenia patients (35). It is noteworthy that *Toxoplasma gondii* infection has been shown to affect the gut microbiome in mice (36). This suggests that the immune and neurochemical disruptions may be partially mediated through gut-related pathways. This interplay highlights the intricate interactions between genetic and environmental factors in disease development (37).

**Table 1:** Clinical evidence for the association between infectious pathogens and schizophrenia.

Category	Antigen	Antibody	Conclusion	Relevance(P<0.05%)	References
Microbial antigen-antibody	Toxoplasma antigen	IgG	The positive rate of anti-Toxoplasma gondii IgG antibodies in patients with SZ is higher than that in the general population, and Toxoplasma gondii infection is associated with higher levels of IgG and an increased incidence of SZ.	Related	(18–20)
		IgM	There was no statistically significant difference in the positive rate of IgM antibodies against Toxoplasma gondii between SZ patients and the general population.	Unrelated	(18, 21)
	Chlamydia pneumoniae antigen	Antibody to Chlamydia pneumoniae	The incidence of Chlamydia infection is significantly increased in SZ patients.	Related	(22)
	Chlamydia trachomatis antigen	Antibody to Chlamydia trachomatis	The IgG antibody titer for Chlamydia trachomatis is significantly elevated in SZ patients.	Related	(23, 24)
	Chlamydia psittaci antigen	Antibody to Chlamydia psittaci	The incidence of Chlamydia psittaci infection is significantly increased in SZ patients.	Related	(25)
	HERV-W Antigen	HERV-W	HERV-W-env is upregulated in SZ patients.	Related	(26, 27)
	HHV-2 Antigen	HHV-2	The anti-HSV2 IgG antibody is significantly increased in schizophrenic patients, among which the HERV-W envelope protein or RNA shows the strongest correlation with SZ.	Related	(28)
	BDV Antigen	BDV	There is an association between BDV infection and the risk of developing SZ.	Related	(29)

TG/TPO: Thyroglobulin/Thyroid Peroxidase; ANAs: Antinuclear Antibodies; HERV-W-env: Human endogenous retrovirus W; HHV-2: Human Herpesvirus 2; BDV: Borna Disease

In fact, the total number of non-redundant genes encoded by the microbiota far exceeds that of the human host, and the composition and function of the microbiota are significantly influenced by environmental factors (38, 39). This genomic feature highlights the potentially critical role of the microbiome in mediating gene-environment interactions in humans.

A growing number of studies indicate that levels of natural antibodies against Gram-negative bacteria and lipopolysaccharide (LPS), such as IgA, IgM, and IgG, are elevated in patients with SZ. The production of these antibodies may be related to gut microbiota dysbiosis and increased intestinal permeability (40). Recent research has shown that gut microbes and their metabolites can enter the systemic circulation and affect the central nervous system, which may be closely related to the mechanisms underlying the symptoms of schizophrenia. Together, this evidence positions the Microbiota-Gut-Brain (MGB) axis as a key pathway through which environmental and immune factors contribute to the pathophysiology of schizophrenia, a premise we will explore in detail throughout this review.

## 2 The microbiota-gut-brain axis and schizophrenia: evidence from clinical and preclinical studies

During fetal development, microbial communities are already present in the maternal placenta, amniotic fluid, and umbilical cord (41–43). The perinatal period represents a critical window for the development of both the gut microbiota and the brain. Factors such as perinatal maternal stress, mode of delivery, environmental exposures, and genetic factors significantly influence the composition of the offspring’s gut microbiota and can alter behavior and Central nervous system (CNS) structure (41–50). Historically, a complex bidirectional communication system between the gastrointestinal tract, its microbiota, and the CNS—comprising neural, hormonal, and immune pathways—has been recognized and termed the MGB axis (51).

In recent years, numerous clinical studies have demonstrated that patients with schizophrenia exhibit characteristic gut dysbiosis, manifested as significant disruptions in microbial relative abundance and reduced α-diversity (52–57). Specifically, these alterations include an increased abundance of *Proteobacteria* and *Lactobacillus*, along with decreased levels of anti-inflammatory commensals such as *Prevotella (*58). This dysbiosis reflects a pro-inflammatory state within the gastrointestinal tract, characterized by an elevated abundance of *Lachnoclostridium* and reduced levels of short-chain fatty acid-producing *Blautia* spp. and *Ruminococcus* spp. (59). These microbial changes correlate with higher lipopolysaccharide (LPS) and lower superoxide dismutase-1 levels (59, 60), which can lead to microbial translocation, systemic inflammation, and increased permeability of both the intestinal and blood-brain barriers (BBB), creating a vicious cycle (61–65). These alterations are associated with specific SZ phenotypes, symptom severity, and treatment response (39, 66–68). For instance, a meta-analysis by Murray et al. indicated that abnormal proliferation of specific microbial taxa (e.g., *Bifidobacterium and Lactobacillus*) is significantly correlated with core clinical features, including worsened negative symptoms, metabolic pathway disturbances, and reduced cortical gray matter volume (57).

## 3 Microbial metabolites in SZ pathology: BBB disruption and neuroinflammation

The alterations in microbial composition described above are closely linked to functional disturbances in the gut ecosystem of patients with schizophrenia. Influenced by the altered gut microbiota composition, SZ patients show an increased incidence of gastrointestinal barrier dysfunction, food antigen sensitivity, inflammation, and metabolic syndrome (56, 69, 70). This may result from severe disruption of functional capacity in the gut microbiota of SZ patients, leading to a marked increase in pro-inflammatory metabolites and a significant decrease in anti-inflammatory metabolites (71). In preclinical and clinical metabolomics studies, approximately 10 metabolites have been identified to exhibit significant alterations in patients with SZ. These include N-acetylaspartate, lactate, tryptophan, kynurenine, glutamate, creatine, linoleic acid, D-serine, glutathione, and 3-hydroxybutyrate, among others (72). A recent study found that elevated levels of lactate and cortisol in the peripheral blood of SZ patients were significantly correlated with decreased immune parameters (such as reactive lymphocytes) and altered concentrations of cerebral metabolites like glutamate and N-acetylaspartate (73). Another study further identified correlations between specific gut bacteria (e.g., Streptococcus-Sobrinus) and metabolites (such as 7-aminomethyl-7-carbaguanine and vitamin D2), and discovered that these microbial features were closely associated with patients’ cognitive function (74). Furthermore, the gut microbiome is associated with changes in brain structure and function in SZ patients, as evidenced by neuroimaging studies linking microbial α-diversity to alterations in gray matter volume and regional homogeneity (75). These alterations in brain structure may facilitate the entry of toxic substances and peripheral inflammatory mediators into the brain, thereby triggering neuroinflammation. This process can involve gut microbiota metabolites crossing the blood-brain barrier, modulating the microbiota-gut-brain axis, and regulating microglial activity and cytokine release.

### 3.1 Bile acids

The potential role of bile acids (BAs) metabolism dysregulation in SZ is likely mediated through interactions with the gut-brain axis (76). Clinical studies have confirmed significant alterations in both the gut microbiota composition and BAs profiles in SZ patients (77), highlighting a potential correlation between BAs dysregulation and the disorder. A metagenome-wide association study further identified gut bacteria unique to SZ patients, including Alkalibacterium, Enterococcus faecium, and Lactobacillus fermentum (78). Cross-sectional studies found that certain gut microbes such as Collinsella, Corynebacterium, Lactobacillus, and Succinivibrio (67); Lachnospiraceae (79); and Veillonella (75) were positively correlated with the severity of SZ. This association between gut microbiota dysbiosis and SZ severity might be mediated through abnormalities in the BAs decoupling process. Metabolomic analysis of serum BAss in SZ patients revealed significantly altered BAs profiles compared to healthy controls (80). Notably, levels of primary Bas, such as CDCA, were generally elevated in patients, whereas levels of secondary Bas (e.g., DCA and LCA) were reduced (80). These altered BAs profiles may subsequently affect the composition of the gut microbiota, thereby interfering with neural function and contributing to the development and progression of SZ. A recent case report described a 39-year-old Persian male with treatment-resistant SZ who, after receiving 300 mg UDCA daily for 12 weeks, showed significant improvement in both positive and negative symptoms, along with enhanced cognitive abilities. Importantly, UDCA treatment was not only effective but also well-tolerated, with no adverse reactions reported during the treatment period, underscoring the safety and efficacy of UDCA supplementation (81). Animal studies have revealed that secondary bile acids may impair the integrity of the BBB. Among these, DCA has been demonstrated to damage endothelial tight junctions, leading to increased BBB permeability (82). This disruption allows toxic substances and peripheral inflammatory mediators to enter the brain, triggering neuroinflammation.

### 3.2 Polyamines

Polyamines (PAs) are important metabolites produced by gut bacteria. Research has confirmed that the gut microbiota is a significant source of polyamine synthesis, with its production of putrescine, spermidine, and spermine playing a key role in maintaining intestinal polyamine homeostasis (83). Historically, PAs were implicated in the etiology of schizophrenia because certain antipsychotic and antimalarial drugs contain structural components resembling spermidine and were associated with extrapyramidal symptoms and psychosis (84). A multi-omics analysis revealed significant disturbances in the polyamine biosynthesis pathway (85). Currently, elevated blood concentrations of PAs (primarily spermine and/or spermidine) have been observed in cases across various subtypes of schizophrenia (86–88). However, data on PA concentrations in the brain remain limited. One study on human brain tissue found no difference in polyamine levels in the frontal cortex or hippocampus of schizophrenia patients compared to control subjects (89). Another study reported significantly elevated agmatine concentrations in both the plasma and postmortem frontal cortex tissue of first-episode and chronic schizophrenia patients (90–93), and found that antipsychotic treatment could reduce blood agmatine levels (94). Elevated spermidine and total PA concentrations were also detected in fibroblasts obtained from SZ patients (95). Furthermore, studies on serum from SZ patients showed elevated levels of polyamine oxidase (the enzyme responsible for degrading PAs) (96, 97), while the activities of three enzymes involved in PA synthesis—ornithine aminotransferase, antizyme inhibitor 1 (AZIN1), and ornithine cyclodeaminase —were found to be reduced in the prefrontal cortex of both treated and untreated patients (98), ultimately potentially leading to disrupted polyamine homeostasis. A translational convergent functional genomics study identified the gene AZIN1, which encodes AZIN1, as a candidate gene for schizophrenia (99), providing genetic support for these findings. The pathophysiological role of PAs in SZ is primarily thought to be mediated through the dopamine pathway and by altering the function of the N-methyl-D-aspartate receptor (100), although the precise mechanisms remain unclear.

Under pathological conditions such as cerebral ischemia and trauma, stress states can induce significant disruption in polyamine metabolism, particularly the abnormal accumulation of putrescine. This alteration is closely associated with vasogenic edema and the disruption of the BBB (101). Research has confirmed that inhibiting polyamine biosynthesis with the ornithine decarboxylase inhibitor α-difluoromethylornithine significantly mitigates increased BBB permeability, Evans blue extravasation, and brain tissue water content in models of cerebral ischemia (102). Notably, this protective effect can be reversed by the administration of exogenous putrescine. This indicates that upregulation of endogenous polyamines, especially putrescine, serves as a critical mediator in BBB injury.

### 3.3 Short-chain fatty acids

The structural integrity of the BBB is maintained by tightly joined brain endothelial cells, astrocytic end-feet, pericytes, and various transport proteins, which stringently regulate the penetration of substances from the blood into the brain tissue (103). SCFAs—such as acetate, propionate, and butyrate—produced by gut microbial fermentation, can enter the systemic circulation, reach the brain, and are present in considerable concentrations in the cerebrospinal fluid (104).

Recent research has shown that SCFAs play a crucial role in modulating the structure and function of the BBB. On the one hand, SCFAs can enhance BBB integrity by regulating the expression of tight junction proteins. For instance, studies in germ-free or antibiotic-treated mouse and rhesus monkey models have demonstrated reduced levels of BBB tight junction proteins (e.g., claudin-5, occludin) and increased BBB permeability; colonization with SCFA-producing strains (such as Clostridium tyrobutyricum and Bacteroides) reversed these effects (105–108). On the other hand, SCFAs can protect the BBB directly or indirectly through anti-inflammatory mechanisms. SCFAs can interact with free fatty acid receptor 3 present on brain endothelial cells (109), suppress inflammatory responses, and alleviate oxidative stress, thereby promoting BBB stability. For example, propionate exerts anti-inflammatory and antioxidant effects by reducing cell surface CD14 expression and influencing the translocation of Nuclear Factor Erythroid 2-Related Factor 2 (110). Peripherally, certain SCFAs (e.g., acetate, propionate, and butyrate) have also been identified to possess anti-inflammatory properties (111–113).

## 4 Gut microbiota dysbiosis and abnormal activation of the innate immune system

### 4.1 Evidence for the association between complement components and schizophrenia

Current studies have identified elevated transcript levels of complement components (C1qA, C3, C4, C5) in patients with schizophrenia (114) (see **Table 2**), suggesting, albeit not entirely consistently, that increased activation of the classical complement pathway may be associated with SZ.

**Table 2:** Evidence for the association between complement components and SZ.

Complement	Study subpopulations	Control populations	Results	References
C1q	Mothers whose offspring developed mental illness in adulthood	Mothers whose offspring did not develop mental illness	The levels of C1q IgG(↑*)	(115)
	FES	Healthy People	Serum C1q levels(↓*)	(116)
	Patients with SZ	Healthy People	The levels of C1q mRNA(↑*)	(117)
	FES	Healthy People	Plasma levels of C1q (↑*)	(118)
	FES	Healthy People	Serum C1 levels(↑*)	(119)
	FES	Healthy People	C1q mRNA and protein (↓*)	(120)
	FES&UHR for psychosis	Healthy People	Serum C1q levels (→)	(121)
	SZ cases with high inflammation	Healthy People	C1qA mRNA(↑*)	(114)
C3	FES	Healthy People	Serum C3 levels (↑*)	(119)
	Patients with SZ	Healthy People	Serum C3 levels (↓*)	(122)
	Patients with SZ	Healthy People	The levels of C3(↑*) in both CSF and plasma	(123)
	FES	Healthy People	Serum C3 levels (→)	(124)
	Patients with SZ	Healthy People	Serum C3 levels C3(↑*)	(125)
	Patients with SZ	Healthy People	C3 mRNA(→)	(117)
	SZ cases with high inflammation	Healthy People	C3 mRNA(↑*); C3 protein(→)	(114)
	FES	Healthy People	Serum C3 levels (↓*)	(116)
	Female subjects with high C3 concentrations	Female subjects with normal C3 concentrations	Serum C3 levels (↓*)	(126)
	Patients with SZ	Healthy People	Serum C3 levels(↑*)	(127)
	FES	Healthy People	The expression of C3(↑*) Serum C3 levels(↑*)	(128)
	Patients with SZ	Healthy People	Serum C3-ana levels (↑)	(129)
	UHR&FES&CSZ	Healthy People	In serum:UHR:C3(↑*) FEP&CSZ:C3(→)	(121)
	Patients with SZ	Healthy People	The frequency of the C3 gene allele(↑*)	(130)
	Patients with SZ	Healthy People	Serum C3 levels(↑*)	(131)
	FES	Healthy People	Plasma levels of C3(→)	(118)
C4	Patients with SZ	Healthy People	C4 mRNA(↑)	(117)
	Patients with SZ	Healthy People	The expression of C4A(↑)	(132)
	Patients with SZ	Healthy People	Serum C4-ana levels(↑*), C4A mRNA(↑*)	(129)
	Mice with overexpressed C4A gene	Mice with normal C4A gene expression.	The expression of C4A(↑*)	(133)
	Mice with overexpressed C4A gene	Mice with normal C4A gene expression.	The expression of C4(↑*)	(134)
	Patients with SZ	Healthy People	Serum C4 levels(↑*)	(125)
	FES	Healthy People	The expression of C4(↓*)	(116)
	FES	Healthy People	The expression of C4(↑*) Plasma levels of C4(↑*)	(118)
	UHR&FES&CSZ	Healthy People	In serum:CSZ&UHR:C4(↑*) FES:C4(→)	(121)
	FES	Healthy People	Serum C4 levels(↑*)	(128)
	Patients with SZ	Healthy People	Serum C4 levels(↑*)	(127)
	The C4 concentration in newborns with symptoms of mental disorders	The normal range of C4 concentrations in healthy newborns	Serum C4 levels(→)	(126)
	FES	Healthy People	Serum C4 levels (↑*)	(119)
	SZ cases with high inflammation	Healthy People	C4 mRNA(↑*) C4 protein (↓*)	(114)
	Patients with SZ	Healthy People	The expression of C4A(↑*)	(120)
	FES	Healthy People	C4 gene mRNA expression(→)	(135)
	FES	Healthy People	C4 levels were elevated(↑*)in the dorsolateral prefrontal cortex and parietal cortex, while remaining unchanged(→) in peripheral tissues	(136)
	YASZ&AOSZ	Healthy People	The expression of C4A(↑)	(137)
	Male patients with SZ or SZ with affective disorder	Female patients with SZ or SZ with affective disorder	In males:C4A(↑*);In females: C4B(↑*)	(138)
	Adult schizophrenic patients	Healthy People	Serum C4 levels (↑*)	(139)
	FES	Healthy People	CSF C4(↑*)	(140)
	FES	Healthy People	Serum C4 levels (↓*)	(124)
	Patients with SZ	Healthy People	The levels of C4(↑*) in both CSF and plasma	(123)
	Male patients with SZ	Female patients with SZ	In protein, serum and CSF levels: C4 allele is stronger in males than in females	(123)
C5	Patients with SZ	Healthy People	CSF C5(↑*)	(141)
	FES	Healthy People	Serum C5 levels(↓*)	(116)
	Patients with SZ	Healthy People	Serum C5-ana levels (→)	(129)

FES: First Episode-SZ; UHR: Ultra-High Risk; CSZ: Chronic SZ;YASZ: Young adult-onset SZ; AOSZ: Adolescent-Onset SZ; CSF: Cerebrospinal fluid

Symbols: ↑*/↓* indicates a significant upward/downward trend (*p* < 0.05); ↑/↓ indicates a gradual upward/downward trend (*p*>0.05); → indicates No trend/Stable.

### 4.2 Gut microbiota dysbiosis and abnormal activation of complement C4

Genetic susceptibility to schizophrenia is significantly associated with polymorphisms in genes related to the complement system, largely attributable to alleles of the complement C4 gene located within the major histocompatibility complex region on chromosome 6 (132). The C4 allele demonstrates the strongest association with SZ risk (132)and is closely linked to increased synaptic phagocytosis and elimination by microglia (8). Furthermore, recent research has found that C4 gene overexpression triggers impaired GluR1 trafficking through an intracellular mechanism involving the endosomal protein SNX27, leading to pathological synaptic loss.

Pathogen exposure has long been recognized as a risk factor for the development of schizophrenia (142). Immune-related environmental variables, such as pathogen infection and gut microbiota dysbiosis, may interact with complement system dysfunction (143). Particularly noteworthy is the finding that genetic polymorphisms of complement components C4A and C4B are significantly associated with various microbial environmental variables in schizophrenia patients, including a history of pathogen exposure and gut ecological imbalance (143). Among these, a negative correlation was observed between plasma lipopolysaccharide-binding protein (LBP) levels and C4A gene copy number was observed in patients with schizophrenia, but not in healthy controls (143). This discovery is significant because LBP, a marker of bacterial translocation, reflects the host’s response to gut microbial translocation and circulating bacterial LPS (144). Together, these results suggest that the complement system, particularly C4A, may play a key role in the gene-environment interactions of schizophrenia by modulating the gut microbiota and systemic immune activation status.

Furthermore, the negative correlation between C4A and C4B copy numbers in SZ patients may reflect a non-random association between these loci. Further analysis revealed that C4A and C4B haplotypes are significantly associated not only with the diagnosis of schizophrenia and environmental factors but also with psychiatric symptoms and cognitive function (143). While multiple previous studies have confirmed the association between the C4A gene and an increased risk of schizophrenia (132), the intrinsic mechanism underlying the negative correlation between C4B and schizophrenia risk remains unclear. It is currently unknown whether a lower C4B copy number has a protective effect against the disease or merely reflects an associated pathological protein deficiency. The negative correlations between C4A and C4B, as well as between C4L and C4S copy numbers, more likely indicate that these loci reside at opposing ends of a risk (C4A and C4L) and protective (C4B and C4S) spectrum, existing in a state of linkage disequilibrium (143). Biochemically, C4A exhibits higher affinity for amino groups, whereas C4B binds more readily to hydroxyl groups, suggesting that C4A may be more involved in binding immune complexes and protein antigens, while C4B plays a more important role in binding carbohydrate-rich microbial antigens (145). Consequently, a deficiency in C4B protein in patients may impair its ability to bind microbial antigens. Conversely, another possibility is that a lower C4B copy number might protect the stability of the host microbiome to some extent by reducing the excessive clearance of beneficial microbes mediated by C4B. A study on pediatric inflammatory bowel disease indicated that individuals with low C4B copy numbers had milder inflammation and higher gut microbial diversity compared to those with high C4B copy numbers (146).

As research on the gut microbiome in schizophrenia expands, integrating C4 genotyping with microbiome analysis is of great importance. Furthermore, these haplotype associations require further analysis to exclude nearby HLA gene variants—which may influence the observed immune phenotype due to linkage disequilibrium with specific C4 alleles (123).

## 5 Potential pathophysiological mechanisms of toll-like receptors and gut bacterial translocation in schizophrenia and psychiatric disorders

Toll-like receptors (TLRs) coordinate the activation of innate immune responses alongside the complement system and play a significant role in the neuroimmune mechanisms of schizophrenia. Studies indicate that activation of TLR2/3/4/5 triggers the NF-κB/NLRP3 pathway and the PI3K/Akt/mTORC1 signaling pathway, leading to microglial activation, neuroinflammation, and neuronal damage (147, 148). Furthermore, thickness changes in the limbic system and cortical brain regions of schizophrenia patients are correlated with abnormal expression of specific TLRs, suggesting their involvement in brain structural remodeling (149). Stimulation of whole blood cells from SZ patients with selective TLR agonists results in enhanced release of pro-inflammatory cytokines (including IL-1β, IL-6, IL-8, and TNF-α) (150), further supporting the role of TLRs in regulating neuroinflammation in schizophrenia.

TLR4, a key receptor for recognizing pathogen-associated molecular patterns such as LPS, activates downstream signaling through both MyD88-dependent and independent pathways, inducing the production of various cytokines and participating in the regulation of neuroinflammation and cellular function (151, 152). In recent years, studies on SZ bodily fluids have consistently reported increased numbers of TLR4-positive monocytes and elevated TLR4 expression in the peripheral blood of schizophrenia patients (149, 153–155), suggesting that TLR4 upregulation is a key factor in the immunopathological process of schizophrenia. Postmortem results show increased TLR4 protein expression in the prefrontal cortex of schizophrenia patients (152), which is associated with activation of the MyD88 and NF-κB pathways (156). Recent research has identified the TLR4/MyD88/NF-κB pathway as playing a central role in various neurological disease models, suggesting its relevance in schizophrenia pathogenesis. For instance, regulatory T cells modulate neuroinflammation and microglial pyroptosis in LPC-induced demyelination via this pathway, thereby alleviating myelin loss and cognitive dysfunction (157). Additionally, TLR4 regulates hippocampal neurogenesis and synaptic function through this pathway, and is involved in neuroinflammation and neuronal apoptosis (158). Conversely, inhibiting TLR4 can suppress microglial activation, alleviate neuroinflammation, improve cognitive function, and mitigate synaptic plasticity impairments and depression-like symptoms via this pathway (159, 160).

The pathological processes described above are often initiated in schizophrenia by a breach of the intestinal barrier, creating a conduit for gut-derived immune activation. Alterations in the microbial flora, gut inflammation, increased intestinal barrier permeability (forming a “leaky gut”), bacterial translocation, and exposure to stressful environments in schizophrenia (144, 152, 156), may trigger innate immunity via TLR4 stimulation. The specific mechanism may involve a compromised intestinal barrier allowing antigens from the gut microbiota to penetrate and contact IgG antibodies, forming immune complexes that circulate in the bloodstream, including to the choroid plexus of the CNS. The chronic accumulation of these complexes can initiate inflammation and contribute to the progression of chronic disease. When a “leaky gut” is present, intestinal barrier permeability is increased, pro-inflammatory substances like LPS may activate inflammatory pathways, be recognized and activated by the TLR4 receptor, and mediate inflammatory responses. This indirect influence of gut microbes on the innate immune system leads to changes in the circulating levels of pro- and anti-inflammatory cytokines, subsequently directly affecting brain function (161).

Exposure to microbial products such as LPS, which can translocate from a leaky gut, activates TLR4 signaling, which is closely intertwined with gut-brain axis interactions. This is corroborated by numerous animal models. Pretreatment with paliperidone (an atypical antipsychotic drug) inhibits TLR4 activation and neuroinflammatory responses in the prefrontal cortex of stressed rats. The mechanism involves modulating stress-induced gut inflammation and reducing plasma LPS levels, thereby influencing brain TLR4 signaling pathways. This result suggests that the therapeutic effects of paliperidone extend beyond its impact on dopamine and serotonin neurotransmission systems (162). Another rat study found that acute restraint stress can upregulate TLR4 gene expression in the frontal cortex by inducing gut microbiota translocation, while intervention with antibiotics or the TLR4 specific inhibitor TAK-242 effectively suppresses this process and reduces the accumulation of inflammatory and oxidative/nitrosative mediators (163). Furthermore, inhibiting TLR4 can also modulate gut microbiota homeostasis and the MyD88/NF-κB axis in ulcerative colitis (164), indicating that TLR4 acts not only as a key sensor of innate immunity but may also regulate neuroimmune crosstalk and gut microenvironment homeostasis. Given that SZ patients also experience loss of gut microbiota homeostasis, targeting the TLR4 signaling pathway and focusing on bacterial translocation and microbiota may offer new avenues for immunomodulatory therapy in schizophrenia.

Specifically, in the “leaky gut” state, gut microbiota dysbiosis occurs, the expression of intestinal epithelial junction proteins (e.g., tight junction proteins) decreases, and intestinal permeability increases, forming a “leaky gut” (165). This breach facilitates bacterial translocation, as evidenced by the significant increase in markers such as soluble CD14 and LBP in the blood of individuals with schizophrenia (144, 166). Upon entering the systemic circulation, microbial products like LPS are recognized by and activate the TLR4 receptor on innate immune cells. This triggers downstream signaling pathways (e.g., NF-κB and MAPK), driving the massive release of pro-inflammatory cytokines, including IL-1β, IL-6, and TNF-α (167–170). These peripheral inflammatory mediators can, in turn, compromise the blood-brain barrier and activate central immune cells, thereby linking gut-derived innate immune stimulation to the neuroinflammation characteristic of schizophrenia. This cascade establishes a self-perpetuating vicious cycle, wherein systemic inflammation exacerbates gut barrier dysfunction and dysbiosis, which then further fuels the inflammatory response.

## 6 Gut microbiota dysbiosis and cytokine abnormalities

### 6.1 Evidence for the association between cytokines and schizophrenia

Given the dysfunction of the BBB in patients with schizophrenia, changes in cytokine levels in the periphery (peripheral blood) and the center (cerebrospinal fluid, CSF) are valuable for assessing the CNS inflammatory state. Numerous studies have identified abnormal alterations in various cytokines in the CSF and peripheral blood of SZ patients (see **Table 3**), most of which are pro-inflammatory cytokines (IL-1β, IL-2, IL-8, TNF-α, and IFN-γ) (188–191), with a smaller proportion being anti-inflammatory cytokines (including TGF-β1, IL-4, and IL-10) (192). The prevailing research view is that a dynamic imbalance between pro-inflammatory and anti-inflammatory cytokines may contribute to the pathogenesis of schizophrenia and subsequent psychopathological symptoms (193–196).

**Table 3:** Summary of meta-analysis of cytokines in peripheral blood and cerebrospinal fluid of SZ patients.

Sample Type	Study subpopulations	Control population	Cytokine evaluated	References
Peripheral Blood	SZ after treatment	Healthy People	IL-6(↑*), IL-6R(↓*)	(171)
	FES&Recurrent SZ	Healthy People	IL-8(↑*)	(172)
	CSZ&FES	CSZ&FES after treatment with risperidone and clozapine	CSZ after treatment with risperidone :IL-6,TNF-α,IL-1β(↓*); FES after treatment with risperidone :IL-6,TNF-α,IL-1β(→); CSZ&FES after treatment with risperidone and clozapine(→)	(173)
	CSZ&FES	Healthy People	CRP/hsCRP,IL-4,IL-6,IL-8,IL-10,TNF-α(↑*)	(174)
	ASZ&CSZ	Healthy People	ASZ: IL-1β,IL-1RA,sIL-2R,IL-6,IL-8,IL-10,TNF-α,IFN-γ(↑*) IL-2,IL-4,IL-12(↑)	(175)
			CSZ: IL-1β,IL-1RA,sIL-2R,IL-6,IL-8,IL-10,TNF-α(↑*) IL-2,IL-4,IL-12,IFN-γ(↓)	
	FES	Healthy People	IFN-γ,IL-6,IL-17,IL-12(↑*)	(176)
	FES(Post-AT)	FES(Pre-AT)	IL-6,TNF-α(↓*); BDNF(↑*)	(177)
	FES	Healthy People	BDNF,NGF(↓*) IL-6,IL-8,TNF-α(↑*)	(178)
	High-risk converters	Non-converters	IL-12(↓)	(179)
	High-risk psychosis	Healthy People	IL-6(↑*),IL-1β(↓*)	
	FES&ESZ	Healthy People	IL-6,TNF-α(↑*)	(180)
	FES(Post-AT)	FES(Pre-AT)	IL-1 β,IL-6,IL-4(↓*)	(181)
	ASZ(Post-AT)	ASZ(Pre-AT)	IL-1β,IL-6,TNF-α,sIL-6R,IFN-γ(↓*)	
	TRS(Post-AT)	TRS(Pre-AT)	IL-6(↑*)	
	All SZ patients(Post-AT)	All SZ patients(Pre-AT)	IL-1β,TNF-α,IFN-γ (↓*); IL-6,TNF-α(↓); sTNF-R2,sIL2-R(↑*)	
	Mothers with offspring affected by SZ	Healthy mothers	CRP,IL-8,IL-10(↑*)	(182)
	FES(4 weeks Post-AT)	FES(Pre-AT)	IL-2,IL-6,IL-1(↓*)	(183)
	ASZ	Healthy People	IFN-γ,IL-1RA,IL-1β,IL-6,IL-8,IL-10,IL-12,sIL-2R,TGF-β,TNF-α(↑*);IL-4(↓*);	(184)
	Acute exacerbation of CSZ		IFN-γ,IL-1RA,IL-1β,IL-6,IL-8,IL-12,sIL-2R,TGF-β,TNF-α(↑*);IL-4,IL-10(↓*)	
	CSZ		IL-1β,IL-6,sIL-2R,TNF-α(↑*);IFN-γ(↓*)	
	ASZ(36–75 days post-AT)	ASZ(Pre-AT)	IL-1β,IL-4,IL-6(↓*);IL-12,sIL-2R(↑*)	
	FES	Healthy People	IL-1β,sIL-2R,IL-6,TNF-α(↑*)	(185)
Cerebrospinal Fluid	SZ after treatment	Healthy People	IL-6(↑*)	(171)
	SZ patients	Healthy People	IL-1β,IL-6,IL-8(↑*);sIL-2R (↓*)	(186)
	SZ patients	Healthy People	IL-1β,IL-6,IL-8(↑*)	(187)

FES: First Episode-Schizophrenia; ASZ: Acute Schizophrenia; CSZ: Chronic Schizophrenia; AST: Acute Schizophrenia After Treatment; CHR: Clinical High-Risk for Psychosis; BDNF: Brain Derived Neurotrophic Factor; CRP: C-Reactive protein; hsCRP: hypersensitive C-reactive protein; ESZ: Early Schizophrenia; TRS: Treatment-Resistant Schizophrenia; AT: Antipsychotic Treatment

Symbols: ↑*/↓* indicates a significant upward/downward trend(*p* < 0.05); ↑/↓ indicates a gradual upward/downward trend(*p*>0.05); → indicates No trend/Stable.

According to **Table 3**, a characteristic pro-inflammatory state can be observed in SZ patients, manifested by persistently elevated levels of pro-inflammatory cytokines such as IL-6, IL-1β, and TNF-α in both peripheral blood and CSF, accompanied by a relative deficiency of anti-inflammatory cytokines like IL-10 and TGF-β. This cytokine imbalance is closely related to symptom severity, cognitive deficits, and treatment response. This aligns with current mainstream research; for example, the TLR4/NF-κB/IL-1β signaling pathway is activated in chronic SZ patients (197), and clozapine reduces the expression of pro-inflammatory genes such as *IL-1*β and *IL-6* by inhibiting the TLR4/NF-κB pathway (198). Additionally, IL-8 mediates the migration and survival of neural stem cells and oligodendrocyte progenitor cells early in life, and elevated CSF cytokines IL-1β and IL-6 suggest the possibility of microglial activation (199–201).

Aggregating data from **Table 3** reveals consistent cross-barrier alterations in cytokine levels in both CSF and peripheral blood across different clinical stages (first-episode/FES, acute/ASZ, and chronic/CSZ), which hold clinical translational significance: 1). Significantly elevated levels of IL-1β, IL-6, and IL-8 in FES and ASZ SZ patients suggest they may be state markers for the onset or acute exacerbation of SZ; 2). The dose-dependent decrease in pro-inflammatory cytokine levels (e.g., IL-1β, IFN-γ, IL-6, TNF-α) following antipsychotic treatment (risperidone and clozapine) indicates value for monitoring treatment response; meanwhile, a significant increase in anti-inflammatory cytokine levels (e.g., sIL-2R) was observed in some patients (181, 184), a finding suggesting that antipsychotic drugs may exert anti-inflammatory-like effects.

### 6.2 Potential pathophysiological mechanisms of gut microbiota and metabolite SCFAs in cytokine abnormalities

SCFAs (such as acetate, propionate, and butyrate) activate receptors like FFAR2/3 and Gpr109a and inhibit histone deacetylase (HDAC), collectively suppressing the activation of the NF-κB signaling pathway, thereby downregulating the expression of pro-inflammatory cytokines (202–204). It is well-known that the NF-κB signaling pathway is typically activated in inflammatory and autoimmune diseases (205–207). Interestingly, all three cytokines elevated in SZ patients in **Table 1** (IL-1β, IL-6, and IL-8) are regulated via the NF-κB pathway. Since cytokines can also regulate the activity of tryptophan catabolism in astrocytes and microglia, this finding corresponds to the alterations in the kynurenine pathway observed in the brains of individuals with schizophrenia (186).

SCFAs play an important regulatory role on various immune cells, particularly those central to maintaining immune homeostasis and anti-inflammatory responses, by inhibiting HDAC. For instance, SCFAs can inhibit NF-κB activation and the secretion of inflammatory cytokines like TNF-α in peripheral blood monocytes, neutrophils, and macrophages, thereby mitigating excessive immune responses. In dendritic cells, butyrate and propionate can impede their normal differentiation and induce an immune tolerant phenotype. Furthermore, SCFAs upregulate the expression of Foxp3, a key transcription factor for regulatory T cells, through HDAC inhibition (208, 209), promoting Treg differentiation and suppressing the production of pro-inflammatory cytokines (210). In SZ patients, the abundance of SCFA-producing bacteria is often altered, leading to reduced SCFA levels, which weakens the capacity to suppress inflammation; the abundance of SCFA-producing bacteria also changes post-treatment (211, 212).

### 6.3 The kynurenine pathway: an interactive mechanism linking gut microbiota, immunity, and neurotransmitters

Specific neurotransmitters, such as GABA, dopamine, glutamate, and serotonin (5-HT), are derived from precursors tyrosine and tryptophan, which are transported across the BBB into the CNS and subsequently converted into neurotransmitters (213). Tryptophan is primarily absorbed in the gut and metabolized by the gut microbiota through three downstream pathways: the 5-HT pathway, the kynurenine pathway, and the indole pathway (214). The kynurenine pathway of tryptophan metabolism represents a critical link through which the MGB axis participates in the immunopathology of schizophrenia. In schizophrenia, immune activation upregulates pro-inflammatory cytokines (e.g., IFN-γ, IL-1, TNF-α), which activate indoleamine 2,3-dioxygenase (IDO) via both IFN-γ receptor (IFN-γR)-dependent and independent pathways (e.g., synergistic action of TLR4, IL-1R, TNF-αR) (215). Enhanced activity of IDO enzymes (IDO-1 and IDO-2) shifts tryptophan metabolism towards the kynurenine pathway, leading to increased concentrations of kynurenic acid (KYNA) within the CNS (216–220). Kynurenine (KYN) is transported across the BBB and metabolized by glial cells into KYNA and quinolinic acid (221, 222) ultimately resulting in excessive KYNA production. Acting as an endogenous antagonist, KYNA inhibits NMDA receptor and α7 nicotinic receptor function (223), suppresses glutamate and acetylcholine neurotransmission (223, 224), and leads to an imbalance in the glutamate, dopamine, and acetylcholine systems, thereby affecting neurotransmission, synaptic organization, and brain connectivity (225, 226).

**Figure 2:**
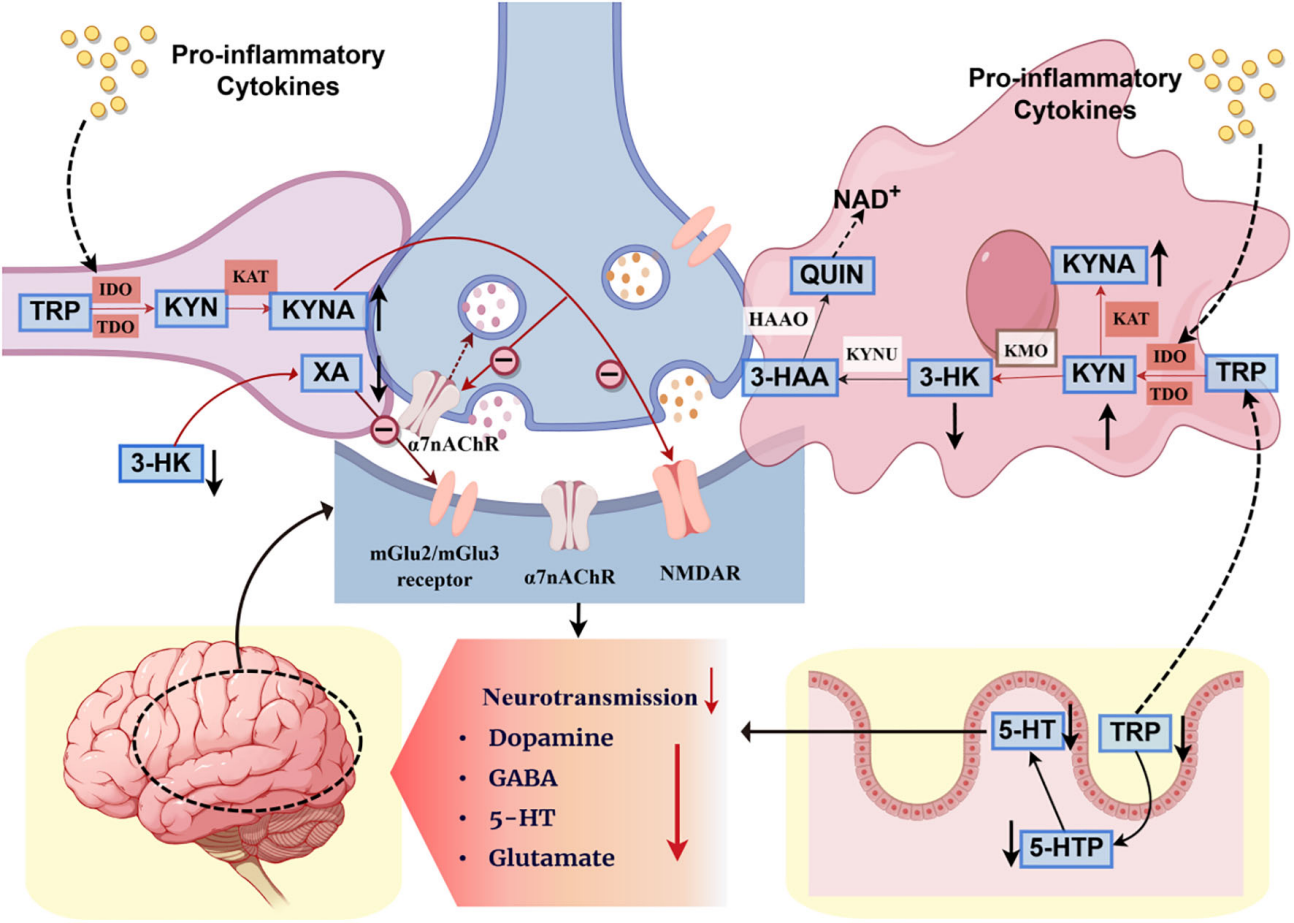
The interactive mechanism of the kynurenine pathway among gut microbiota, immunity, and neurotransmitters. TRP, Tryptophan; KYN, Kynurenine; KYNA, Kynurenic acid; 3-HK, 3-Hydroxykynurenine; QUIN, Quinolinic acid; 3-HAA, 3-Hydroxyanthranilic acid;α7nAChR, α7-nicotinic acetylcholine receptors; NMDAR, N-methyl-D-aspartate receptor; GABA, Gamma-Aminobutyric Acid; 5-HT, 5-Hydroxytryptamine, Serotonin; 5-HTP, 5-Hydroxytryptophan; IDO, Indoleamine 2,3-Dioxygenase; TDO, Tryptophan 2,3-Dioxygenase; KAT, Kynurenine Aminotransferase; KMO, Kynurenine Monooxygenase; 3-HAO, 3-Hydroxyanthranilic Acid Oxygenase; HAAO, 3-Hydroxyanthranilate-3,4-dioxygenase; XA, Xanthurenic acid.

On the other hand, activation of the KP results in substantial tryptophan consumption, reducing the substrate available for 5-HT synthesis (see **Figure 2**). Over 90% of the body’s 5-HT is synthesized in the gut by enterochromaffin cells, which absorb tryptophan from dietary proteins as a substrate for 5-HT synthesis; this process is regulated by SCFAs and the kynurenine synthesis pathway (227, 228). Studies in animal models with gut microbiota depletion have shown increased plasma tryptophan, elevated brain serotonin concentrations, and reduced kynurenine pathway activity, all of which normalize following microbiota restoration (229–231). Gut microbiota dysbiosis and immune activation can further enhance IDO activity, exacerbating the conversion of tryptophan to KYNA. This leads to decreased peripheral and central 5-HT levels, impaired receptor function, reduced concentrations of tryptophan and 5-HT (232), worsened symptoms of affective disorders, elevated levels of tryptophan catabolites (233, 234), and increased concentrations of toxic metabolites in the CNS (235).

Animal experiments have demonstrated that acute tryptophan depletion reduces brain tryptophan concentration by 70%, leading to decreased serotonin levels, diminished 5-HT receptor binding (236, 237), and effects on compulsive behavior (238). Similar results have been observed in human cerebrospinal fluid (CSF) studies (239). This suggests that immune dysregulation-induced gut microbiota dysbiosis and activation of the kynurenine pathway cause acute tryptophan depletion in the gut. This, in turn, leads to reduced brain tryptophan concentration, decreased 5-HT levels, and diminished 5-HT receptor binding, which may underlie the 5-HT system dysfunction, impaired neurotransmission, and negative cognitive effects observed in SZ.

### 6.4 Limitations

This study has several limitations. First, despite conducting an extensive literature search, we cannot entirely exclude the possibility that some published studies may have been overlooked. Second, the multiple meta-analyses and systematic reviews cited in this article may carry a risk of accumulated type I errors (240). Furthermore, although we endeavored to include studies that performed stratified analyses of patients with schizophrenia, the interpretability and generalizability of the results may still be constrained by limited sample sizes and considerable population heterogeneity—such as confounding factors including age, gender, ethnicity, smoking history, and BMI (125).

## 7 Conclusions and discussion

### 7.1 Integrated mechanisms of the microbiota-gut-brain-immune axis in schizophrenia

**Figure 3:**
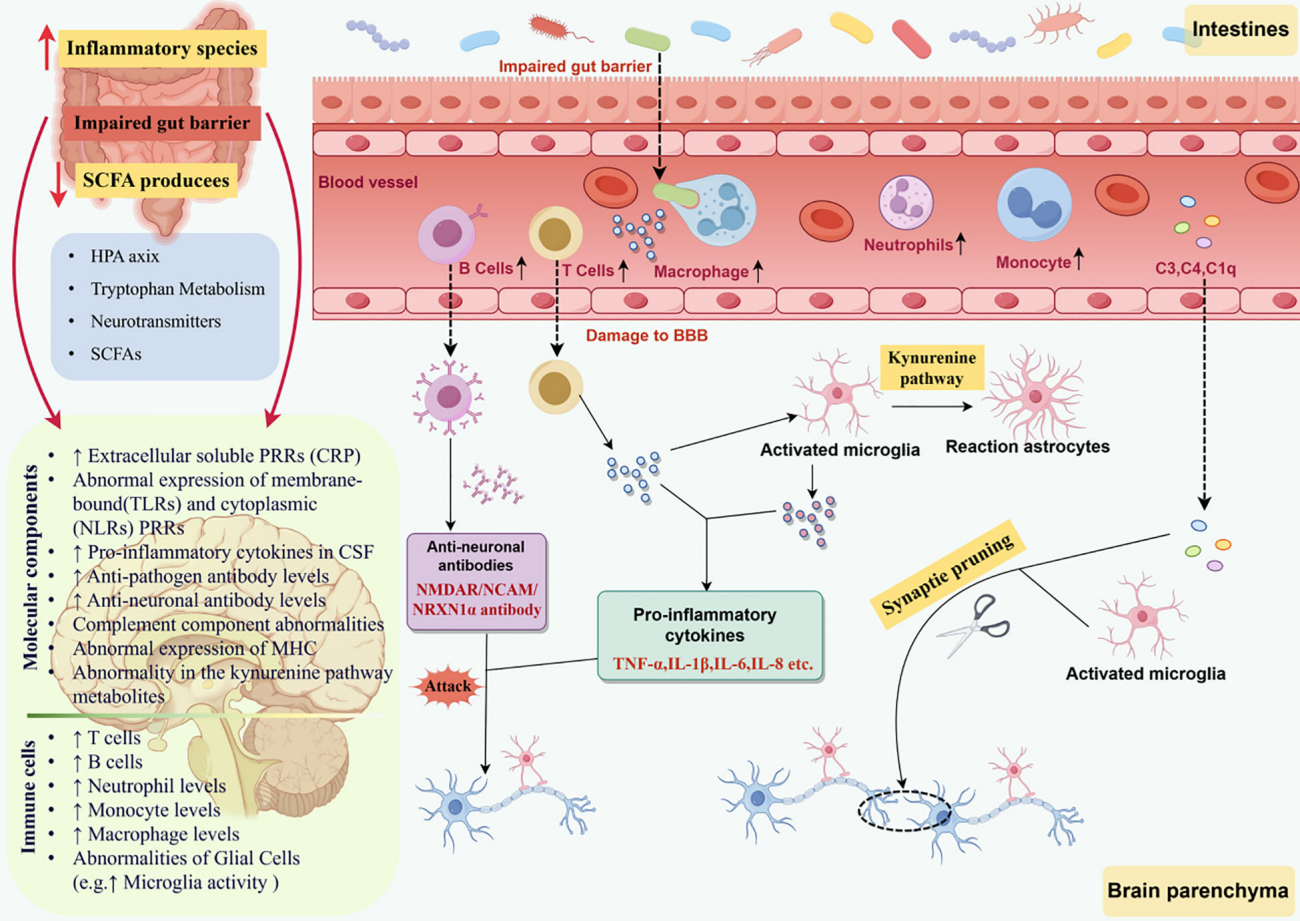
Integrated mechanisms of the microbiota-gut-brain-immune axis in schizophrenia. HPA axis, Hypothalamic-Pituitary-Adrenal Axis; SCFAs, Short-Chain Fatty Acids; PRRs, Pattern Recognition Receptors; CRP, C-Reactive Protein; TLRs, Toll-like receptors; NLRs, Nucleotide-binding Oligomerization Domain-like Receptors; CSF, Cerebrospinal Fluid; MHC, Major Histocompatibility Complex; BBB, Blood-Brain Barrier; C3/C4/C1q, Complement Component 3/4/1q.

By integrating the evidence discussed throughout this review, we propose a comprehensive model illustrating the MGB-immune interactions in schizophrenia (See **Figure 3**). At the peripheral level, gut microbiota dysbiosis and a reduction in microbial metabolites—particularly SCFAs—promote increased antigen exposure and activation of innate immune responses. This leads to the activation of immune cells (e.g., T cells, B cells, NK cells, monocytes/macrophages) and dysfunction of glial cells, culminating in the release of pro-inflammatory cytokines and complement components such as C4. Accompanied by impaired BBB integrity, these peripheral immune factors gain access to the CNS, where they mediate neuroinflammatory responses, resulting in synaptic damage and neuronal dysfunction.

In the CNS, dysbiosis directly or indirectly modulates the function of microglia and astrocytes via metabolites including SCFAs. Aberrantly activated glial cells exacerbate neuroinflammation and influence synaptic pruning processes, with complement C4-mediated synaptic phagocytosis playing a critical role. Concurrently, pro-inflammatory cytokines regulate the activity of key enzymes in tryptophan metabolism—indoleamine IDO and TDO—promoting the production of kynurenine pathway metabolites such as KYNA. This disrupts multiple neurotransmitter systems, including dopamine, GABA, glutamate, and serotonin, further exacerbating neurotransmission dysfunction. Moreover, compromised intestinal barrier integrity and gut-derived microbial components such as LPS activate TLR/NF-κB/NLRP3 pathways, which not only drive systemic inflammation but also modulate gut-derived tryptophan metabolism and serotonin synthesis, thereby coupling immune regulation with neuromodulatory functions.

The gut microbiota, as a critical “microbial organ,” has garnered extensive attention for its role in neuropsychiatric disorders such as SZ. However, the causal relationships among microbial dysbiosis, immune dysregulation, and disease onset remain elusive. It is still unclear whether dysbiosis acts as a driver, a consequence, or both. Elucidating this issue is complicated by multiple confounding factors, including population heterogeneity, medication use, dietary and lifestyle variations, disease staging, and inconsistencies in research methodologies. Although small-scale studies and disparities in technical procedures—such as sample processing, sequencing, and bioinformatic analyses—limit the reliability and generalizability of the findings. Therefore, further large-scale and better-standardized clinical studies with stratified populations are urgently needed to provide more robust data on the association between altered gut microbial features and schizophrenia.

### 7.2 Comparative analysis of gut microbiota in neuropsychiatric disorders

It is well established that the gut microbiota regulates brain function and behavior. The immune-mediated dysregulation of the MGB axis detailed in this review is observed not only in schizophrenia but also across various neurological disorders (241), including neurodevelopmental disorders (242), epilepsy (243), and depression (244), among others. Major depressive disorder is characterized by an elevated Bacteroidetes/Firmicutes ratio (5), accompanied by enrichment of *Bacteroides* and depletion of *Blautia*, *Faecalibacterium*, and *Coprococcus*, alongside increased abundance of *Eggerthella* and elevated levels of pro-inflammatory genera such as *Escherichia (*245). In contrast, anxiety disorders demonstrate an increased *Firmicutes/Bacteroidetes* ratio (246), reduced abundance of SCFA-producing genera including *Bifidobacterium* and *Lactobacillus*, while *Akkermansia* abundance shows a negative correlation with anxiety severity (247, 248). Bipolar disorder similarly exhibits disruption of the *Firmicutes*/*Bacteroidetes* ratio and reduced α-diversity, featuring increased *Streptococcaceae* and *Bacteroidaceae* abundance contrasting with depletion of anti-inflammatory commensals such as *Faecalibacterium (*249).Among neurodegenerative disorders, Parkinson’s disease shows increased abundance of *Lactobacillus* and *Bifidobacterium* with concurrent reduction in *Faecalibacterium*, *Coprococcus*, and *Blautia (*250, 251), where decreased *Blautia* abundance correlates with clinical severity and reduced fecal butyrate levels (252). Alzheimer’s disease manifests through reduced beneficial bacteria including *Bifidobacterium* and elevated opportunistic pathogens such as *Escherichia* and *Clostridium (*253, 254). Autism spectrum disorder(ASD) presents with reduced microbial diversity yet increased biomass in pediatric populations (255, 256), featuring a shift from beneficial microorganisms toward spore-forming, antibiotic-resistant, and/or neurotoxin-producing species (257). Specific alterations include reductions in *Prevotella*, *Coprococcus*, and *Veillonellaceae*, alongside overgrowth of Desulfovibrio (positively correlated with Autism spectrum disorder severity) (258), *Sutterella*, *Ruminococcus*, *Clostridium*, *Megamonas*, and *Candida (*259, 260), accompanied by an elevated *Firmicutes/Bacteroidetes* ratio (255). Notably, *Candida* overgrowth and associated toxin production may exacerbate neurobehavioral symptoms (37). Epilepsy research similarly reveals substantial gut microbiota dysbiosis, with most studies demonstrating reduced α-diversity in treatment-resistant epilepsy (261–264). Phylum-level analyses indicate predominant *Firmicutes* with relative *Bacteroidetes* reduction in some studies (264, 265), while others report increased *Actinobacteria*, *Verrucomicrobia*, or pro-inflammatory *Proteobacteria*, alongside potential reduction of beneficial *Bacteroidetes* and *Actinobacteria (*262, 266).

In summary, these disorders share fundamental microbial alterations: 1) Structural dysbiosis manifested through disrupted *Bacteroidetes*/*Firmicutes* ratios and reduced α-diversity; 2) Functional impairment characterized by universal depletion of anti-inflammatory, SCFA-producing genera including *Faecalibacterium*, *Blautia*, and *Bifidobacterium*; 3) Common pathophysiological mechanisms involving impaired SCFA production, immune-inflammatory activation, and dysregulated neuroactive metabolite metabolism. These alterations promote systemic low-grade inflammation through an imbalance between elevated pro-inflammatory cytokines (e.g., IL-6, TNF-α) and anti-inflammatory mediators (e.g., IL-10) (267–269). This systemic inflammation, akin to the processes described in schizophrenia, can traverse the blood-brain barrier or transmit via vagal afferents, subsequently activating microglia and exacerbating neuroinflammatory processes while impairing prefrontal cortex-mediated executive functions including decision-making and emotional regulation (270). Pro-inflammatory cytokines disrupt neurotransmitter metabolism through mechanisms such as inhibited tryptophan conversion to serotonin while driving microglial activation, thereby amplifying neuroinflammation (271). Activated microglia release chemokines, cytokines, and reactive oxygen species, crucially contributing to neuroinflammatory cascades while disrupting neurotransmitter balance and synaptic plasticity through pro-inflammatory phenotypic transformation. Notwithstanding these commonalities, disorder specific patterns emerge. Major depressive disorder demonstrates *Bacteroidetes*/*Firmicutes* ratio elevation contrasting with anxiety disorders, while ASD presents the distinct profile of reduced diversity with increased biomass and concurrent expansion of multiple opportunistic pathogens. These shared and distinct features collectively illustrate the multifaceted involvement of gut microbiota dysbiosis in neuropsychiatric disorders. Beyond inflammation, psychological stress represents another prevalent pathophysiological feature in microbiota-associated diseases. Stress contributes to depression (272), schizophrenia (273), autism spectrum disorder (274), epilepsy (275), and migraine (276). Significant comorbidity exists among these conditions, exemplified by the frequent co-occurrence of depression and ASD with epilepsy (277), the common comorbidity of depression with migraine (277), and the elevated prevalence of inflammatory bowel disease or irritable bowel syndrome among migraine patients (278).

## 8 Future perspectives

The gut microbiota can influence drug bioavailability and efficacy through metabolism, while most psychotropic medications possess anti-inflammatory properties and may directly alter microbial composition. A recent study revealed that gut microbiome abnormalities in schizophrenia patients were primarily associated with resistance to antipsychotic treatment, whereas this correlation was significantly weaker in patients who responded well to atypical antipsychotics (279). This suggests that structural changes in the gut microbiota may serve more as a potential biomarker for clozapine resistance rather than an intrinsic feature of schizophrenia itself. Underlying mechanisms may involve microbial-mediated drug metabolism, transformation, and modulation of intestinal barrier function.

Furthermore, current immunomodulatory therapies, including immunosuppressants and biologics, remain predominantly palliative. Long-term administration often leads to drug tolerance and opportunistic infections (280). The substantial comorbidity burden—approximately 30% of inflammatory bowel disease patients develop anxiety or depression—highlights the urgent need for dual-effect therapies that simultaneously address intestinal inflammation and gut-brain axis modulation. Combinatorial treatment strategies targeting the gut-brain interplay represent a paradigm shift in managing psychiatric comorbidities in systemic disorders.

By assessing baseline microbiome profiles, it becomes possible to identify individuals at high risk for poor response to relevant medications prior to treatment initiation. This enables the development of personalized dosing regimens or adjunctive microecological interventions (e.g., probiotic/prebiotic supplementation) to enhance therapeutic efficacy while reducing the incidence of drug resistance and adverse effects. These advances underscore the translational value of microbiome research in the precision medicine of mental disorders.

Although microbial-based interventions, such as probiotics, demonstrate therapeutic potential—for instance, certain strains of Lactobacillus and Bifidobacterium have shown preliminary efficacy in alleviating depressive symptoms (281)—their benefits in schizophrenia remain inconsistent and lack high-quality clinical support (282). Therapeutic strategies targeting the MGB axis, including prebiotics, synbiotics, and fecal microbiota transplantation, are not universally applicable. Instead, they should be tailored based on disease subtype, patient stratification, and bacterial functionality. Their clinical utility urgently requires validation through well-designed, large-scale randomized controlled trials.
